# Competency gap among graduating nursing students: what they have achieved and what is expected of them

**DOI:** 10.1186/s12909-024-05532-w

**Published:** 2024-05-16

**Authors:** Majid Purabdollah, Vahid Zamanzadeh, Akram Ghahramanian, Leila Valizadeh, Saeid Mousavi, Mostafa Ghasempour

**Affiliations:** 1 Department of Nursing, Khoy University of Medical Sciences, Khoy, Iran; 2 Medical Education Research Center, Health Management and Safety Promotion Research Institute, Tabriz University of Medical Sciences, Tabriz, Iran; 3 Department of Medical-Surgical Nursing, School of Nursing and Midwifery, Shahid Beheshti University of Medical Sciences, Tehran, Iran; 4 Department of Medical Surgical Nursing, Faculty of Nursing and Midwifery, Tabriz University of Medical Sciences, Tabriz, Iran; 5 Department of Pediatric Nursing, School of Nursing and Midwifery, Shahid Beheshti University of Medical Sciences, Tehran, Iran; 6 Department of Epidemiology and Biostatistics, Assistant Professor of Biostatistics, School of Health, Tabriz University of Medical Sciences, Tabriz, Iran

**Keywords:** Nursing education, Competence, Self-Assessment, Nursing students, Professional Competency Professional

## Abstract

**Background:**

Nurses’ professional competencies play a significant role in providing safe care to patients. Identifying the acquired and expected competencies in nursing education and the gaps between them can be a good guide for nursing education institutions to improve their educational practices.

**Methods:**

In a descriptive-comparative study, students’ perception of acquired competencies and expected competencies from the perspective of the Iranian nursing faculties were collected with two equivalent questionnaires consisting of 85 items covering 17 competencies across 5 domains. A cluster sampling technique was employed on 721 final-year nursing students and 365 Iranian nursing faculties. The data were analyzed using descriptive statistics and independent t-tests.

**Results:**

The results of the study showed that the highest scores for students’ acquired competencies and nursing faculties’ expected competencies were work readiness and professional development, with mean of 3.54 (SD = 0.39) and 4.30 (SD = 0.45), respectively. Also, the lowest score for both groups was evidence-based nursing care with mean of 2.74 (SD = 0.55) and 3.74 (SD = 0.57), respectively. The comparison of competencies, as viewed by both groups of the students and the faculties, showed that the difference between the two groups’ mean scores was significant in all 5 core-competencies and 17 sub-core competencies (*P* < .001). Evidence-based nursing care was the highest mean difference (mean diff = 1) and the professional nursing process with the lowest mean difference (mean diff = 0.70).

**Conclusion:**

The results of the study highlight concerns about the gap between expected and achieved competencies in Iran. Further research is recommended to identify the reasons for the gap between the two and to plan how to reduce it. This will require greater collaboration between healthcare institutions and nursing schools.

## Introduction| Background

Nursing competence refers to a set of knowledge, skills, and behaviors that are necessary to successfully perform roles or responsibilities [1]. It is crucial for ensuring the safe and high-quality care of patients [2–5]. However, evaluating nursing competence is challenging due to the complex, dynamic, and multi factorial nature of the clinical environment [3]. The introduction of nursing competencies and their assessment as a standard measure of clinical performance at the professional level has been highlighted by the Association of American Colleges of Nursing [6, 7]. As a result, AACN (2020) introduces competence assessment as an emerging concept in nursing education [7].

On the other hand, the main responsibility of nursing education is to prepare graduates who have the necessary competencies to provide safe and quality care [3]. Although it is believed that it is impossible to teach everything to students, acquiring some competencies requires entering a real clinical setting and gaining work experience [8]. However, nursing students are expected to be competent to ensure patient safety and quality of care after graduation [9]. To the extent that the World Health Organization (WHO), while expressing concern about the low quality of nursing education worldwide, has recommended investing in nursing education and considers that the future to require nurses who are theoretically and clinically competent [5]. Despite efforts, the inadequate preparation of newly graduated nursing students and doubts about the competencies acquired in line with expectations to provide safe care for entering the nursing setting have become a global concern [10–13]. The results of studies in this field are different. The results of Amsalu et al. showed that the competence of newly graduated nursing students to provide quality and safe care was not satisfactory [14]. Some studies have also highlighted shortcomings in students’ “soft” skills, such as technical competency, critical thinking, communication, teamwork, helping roles, and professionalism [15]. Additionally, prior research has indicated that several nursing students have an unrealistic perception of their acquired competencies before entering the clinical setting and they report a high level of competence [2]. In other study, Hickerson et al. showed that the lack of preparation of nursing students is associated with an increase in patient errors and poor patient outcomes [16]. Some studies also discussed nursing competencies separately; Such as patient safety [17], clinical reasoning [18], interpersonal communication [19], and evidence-based care competence [20].

On the other hand, the growing need for safe nursing care and the advent of new educational technologies, the emergence of infectious diseases has increased the necessity of nursing competence. As a result, the nursing profession must be educated to excellence more than ever before [5, 21, 22]. Therefore, the self-assessment of students’ competence levels as well as the evaluation of nursing managers about the competencies expected from them is an essential criterion for all healthcare stakeholders, educators, and nursing policymakers to ensure the delivery of safe, and effective nursing care [9, 23, 24].

However, studies of nurse managers’ perceptions of the competence of newly graduated nursing students are limited and mostly conducted at the national level. Hence, further investigation is needed in this field [25, 26]. Some other studies have been carried out according to the context and the needs of societies [3, 26–28]. The results of some other studies in the field of students’ self-assessment of perceived competencies and managers’ and academic staff’s assessment of expected competency levels are different and sometimes contradictory, and there is the “academic-clinical gap” between expected and achieved competencies [25, 29, 30]. A review of the literature showed that this gap has existed for four decades, and the current literature shows that it has not changed much over time. The academe and practice settings have also been criticized for training nurses who are not sufficiently prepared to fully engage in patient care [1]. Hence, nursing managers must understand the expected competencies of newly graduated students, because they have a more complete insight into the healthcare system and the challenges facing the nursing profession. Exploration of these gaps can reveal necessities regarding the work readiness of nursing graduates and help them develop their competencies to enter the clinical setting [1, 25].

Although research has been carried out on this topic in other countries, the educational system in those countries varies from that of Iran’s nursing education [31, 32]. Iran’s nursing curriculum has tried to prepare nurses who have the necessary competencies to meet the care needs of society. Despite the importance of proficiency in nursing education, many nursing graduates often report feeling unprepared to fulfill expected competencies and they have deficiencies in applying their knowledge and experience in practice [33]. Firstly, the failure to define and identify the expected competencies in the nursing curriculum of Iran led to the absence of precise and efficient educational objectives. Therefore, it is acknowledged that the traditional nursing curriculum of Iran focuses more on lessons organization than competencies [34]. Secondly, insufficient attention has been given to the scheduling, location, and level of competencies in the nursing curriculum across different semesters [35]. Thirdly, the large volume of content instead of focusing on expected competencies caused nursing graduates challenged to manage complex situations [36]. Therefore, we should not expect competencies such as critical thinking, clinical judgment, problem-solving, decision-making, management, and leadership from nursing students and graduates in Iran [37]. Limited research has been conducted in this field in Iran. Studies have explored the cultural competence of nursing students [38] and psychiatric nurses [39]. Additionally, the competence priorities of nurses in acute care have been investigated [40], as well as the competency dimensions of nurses [41].

In Iran, after receiving the diploma, the students participate in a national exam called Konkur. Based on the results of this exam, they enter the field of nursing without conducting an aptitude test interview and evaluating individual and social characteristics. The 4-year nursing curriculum in Iran has 130 units including 22 general, 54 specific, 15 basic sciences, and 39 internship units. In each semester, several workshops are held according to the syllabus [42]. Instead of the expected competencies, a list of general competencies is specified as learning outcomes in the program. Accepted students based on their rank in the exam and their choice in public and Islamic Azad Universities (non-profit), are trained with a common curriculum. Islamic Azad Universities are not supported by government funding and are managed autonomously, this problem limits the access to specialized human resources and sufficient educational fields, and the lower salaries of faculty members in Azad Universities compared to the government system, students face serious challenges. Islamic Azad Universities must pay exorbitant fees to medical universities for training students in clinical departments and medical training centers, doubling these Universities’ financial problems. In some smaller cities, these financial constraints cause students to train in more limited fields of clinical training and not experience much of what they have learned in the classroom in practice and the real world of nursing. The evaluation of learners in the courses according to the curriculum is based on formative and summative evaluation with teacher-made tests, checklists, clinical assignments, conferences, and logbooks. The accreditation process of nursing schools includes two stages internal evaluation, which is done by surveying students, professors and managers of educational groups, and external accreditation is done by the nursing board. After completing all their courses, to graduate, students must participate in an exam called “Final”, which is held by each faculty without the supervision of an accreditation institution, the country’s assessment organization or the Ministry of Health, and obtain at least a score of 10 out of 20 to graduate.

Therefore, we conducted this comprehensive study as the first study in Iran to investigate the difference between the expected and perceived competence levels of final year nursing students. The study’s theoretical framework is based on Patricia Benner’s “From Novice to Expert” model [43].

## Materials and methods

### AIM

The present study had the following three objectives:

Determining self-perceived competency levels from the perspective of final year nursing students in Iran.

Determining expected levels of competency from the perspective of nursing faculties in Iran.

To determine the difference between the expected competencies from the perspective of nursing faculties and the achieved competencies from the perspective of final-year nursing students.

### Design

This study is a descriptive-comparative study.

#### Sample

First, we obtained a list of all nursing schools in the provinces of Iran from the Ministry of Health (*n* = 31). From 208 Universities, 72 nursing schools were randomly selected using two-stage cluster sampling. Among the selected faculties, we chose 721 final-year nursing students and 365 nursing faculties who met the eligibility criteria for the study. Final-year nursing students who consented to participate in the study were selected. Full-time faculty members with at least 2 years of clinical experience and nurse managers with at least 5 years of clinical education experience were also included. In this study, nursing managers, in addition to their educational roles in colleges, also have managerial roles in the field of nursing. Some of these roles include nursing faculty management, nursing board member, curriculum development and review, planning and supervision of nursing education, evaluation, and continuous improvement of nursing education. The selection criteria were based on the significant role that managers play in nursing education and curriculum development [44]. Non-full-time faculty members and managers without clinical education experience were excluded from the study.

#### Instrument

The instrument used in this study is a questionnaire developed and psychometrically tested in a doctoral nursing dissertation [45]. To design the tool, the competencies expected of undergraduate nursing students in Iran and worldwide were first identified through a scoping review using the methodology recommended by the Joanna Briggs Institute (JBI) and supported by the PAGER framework. Summative content analysis by Hsieh and Shannon (2005) was used for analysis, which included: counting and comparing keywords and content, followed by interpretation of textual meaning. In the second step, the results of the first step were used to create tool statements. Then the validity of the instrument was checked by face validity, content validity (determination of the ratio and index of content validity), and validity of known groups. Its reliability was also checked by internal consistency using Cronbach’s alpha method and stability using the test-retest method. The competency questionnaire comprises 85 items covering 17 competencies across 5 domains: “individualized care” (4 competencies with 21 items), “evidence-based nursing care” (2 competencies with 10 items), “professional nursing process” (3 competencies with 13 items), “nursing management” (2 competencies with 16 items), and “work readiness and professional development” (6 competencies with 25 items) [45]. “The Bondy Rating Scale was utilized to assess the competency items, with ratings ranging from 1 (Dependent) to 5 (Independent) on a 5-point Likert scale [46]. The first group (nursing students) was asked to indicate the extent to which they had acquired each competency. The second group (nursing faculties) was asked to specify the level to which they expected nursing students to achieve each competency.

### Data collection

First, the researcher contacted the deans and managers of the selected nursing schools by email to obtain permission. After explaining the aims of the study and the sampling method, we obtained the telephone number of the representative of the group of final year nursing students and also the email of the faculty members. The representative of the student group was then asked to forward the link to the questionnaire to 10 students who were willing to participate in the research. Informed consent for students to participate in the online research was provided through the questionnaires, while nursing faculty members who met the eligibility criteria for the study received an informed consent form attached to the email questionnaire. The informed consent process clarified the study objectives and ensured anonymity of respondent participation in the research, voluntary agreement to participate and the right to revoke consent at any time. An electronic questionnaire was then sent to 900 final year nursing students and 664 nursing faculties (from 4 March 2023 to 11 July 2023). Reminder emails were sent to nursing faculty members three times at two-week intervals. The attrition rate in the student group was reported to be 0 (no incomplete questionnaires). However, four questionnaires from nursing faculty members were discarded because of incomplete responses. Of the 900 questionnaires sent to students and 664 sent to nursing faculties, 721 students and 365 nursing faculty members completed the questionnaire. The response rates were 79% and 66% respectively.

### Analysis

Data were analyzed using SPSS version 22. Frequencies and percentages were used to report categorical variables and mean and standard deviations were used for quantitative variables. The normality of the quantitative data was confirmed using the Shapiro-Wilk and Skewness tests. An independent t-test was used for differences between the two groups.

## Results

Data analysis revealed that out of 721 students, 441 (61.20%) was female. The mean and deviation of the students’ age was 22.50 (SD = 1.21). Most of the students 577 (80%) were in their final semester. Also, of the total 365 faculties, the majority were female 253 (69.31%) with a mean of age 44.06 (SD = 7.46) and an age range of 22–65. The academic rank of most nursing faculty members 156 (21.60%) was assistant professor (Table 1).

**Table 1:** Characteristics of participants

Variables	Nursing students (*n* = 721)	Faculty members (*n* = 365)
Gender	Frequency (%)	Frequency (%)
Male	280 (38.80%)	112 (30.68%)
Female	441(61.20%)	253 (69.31%)
Educational semester		
Semester 7	144 (20%)	-
Semester 8	577 (80%)	-
Scientific ranking		
Instructor	-	129 (17.90%)
Assistant Professor	-	156 (21.60%)
Associate Professor	-	53 (7.70%)
Full Professor	-	27 (3.70)
Variables(Years)	Mean (SD)	Mean (SD)
Age	22.50 (1.21)	44.06 (7.46)
Clinical experience	-	10.25 (7.01)
Management experience	-	5.07 (3.46)
Educational experience	-	13.94 (7.69)

The results of the study showed that in both groups the highest scores achieved by the students and expected by the nursing faculty members were work readiness and professional development with a mean and standard deviation of 3.54 (0.39) and 4.30 (0.45) respectively. The lowest score for both groups was also evidence-based nursing care with a mean and standard deviation of 2.74 (0.55) for students and 3.74 (0.57) for nursing faculty members (Table 2).

**Table 2:** The Core competencies obtained from the student’s perspective and the expected Core competencies from the nursing faculty member’s perspective (from the highest to the lowest scores)

Expected core competencies from the academic perspective (Nursing Faculty Members)	Mean (SD)	Core competencies obtained from the student’s perspective (Students)	Mean (SD)	Competency Gap in the Core competencies
Work readiness and professional development	4.30 (0.45)	Work readiness and professional development	3.54 (0.39)	0.76
Nursing administration	4.24 (0.42)	Individualized care	3.40 (0.47)	0.84
Individualized care	4.13 (0.44)	Nursing administration	3.39 (0.33)	0.74
Professional nursing process	3.92 (0.50)	Professional nursing process	3.21 (0.52)	0.71
Evidence-based nursing care	3.74 (0.57)	Evidence-based nursing care	2.74 (0.55)	1

Also, the result of the study showed that the highest expected competency score from the nursing faculty members’ point of view was the safety subscale. In other words, faculty members expected nursing students to acquire safety competencies at the highest level and to be able to provide safe care independently according to the rating scale (Mean = 4.51, SD = 0.45). The mean score of the competencies achieved by the students was not above 3.77 in any of the subscales and the highest level of competency achievement according to self-report of students was related to safety competencies (mean = 3.77, SD = 0.51), preventive health services (mean = 3.69, SD = 0.79), values and ethical codes (mean = 3.67, SD = 0.77), and procedural/clinical skills (mean = 3.67, SD = 0.71). The other competency subscales from the perspective of the two groups are presented in Table 3, from highest to lowest score.

**Table 3:** The Sub-Core competencies achieved from the student’s perspective and the expected Sub-Core competencies from the nursing faculty members perspective, in the order of the highest to the lowest score

Core competencies domains	Expected Sub-Core Competencies from the nursing faculty member’s perspective	Mean (SD) for Sub-Core Competence	Core competencies domains	Sub-Core Competencies achieved from the student’s perspective	Mean (SD) for Sub-Core Competence	Competency Gap in the Sub-Core Competencies
(WRPD)	Safety	4.51 (0.45)	(WRPD)	Safety	3.77 (0.51)	0.74
(NAC)	Informatics & documentation	4.48 (0.44)	(WRPD)	Preventative health services	3.69 (0.60)	0.79
(WRPD)	Preventative health services	4.44 (0.63)	(ICC)	Value and ethical codes	3.67 (0.47)	0.77
(WRPD)	Legality	4.38 (0.64)	(WRPD)	Clinical/Procedural skills	3.67 (0.51)	0.71
(ICC)	Cultural humility	4.36 (0.48)	(NAC)	Informatics & documentation	3.63 (0.49)	0.73
(ICC)	Value and ethical codes	4.33 (0.46)	(ICC)	Cultural humility	3.55 (0.70)	0.78
(WRPD)	Clinical/Procedural skills	4.28 (0.54)	(ICC)	Therapeutic communication participatory decision making	3.55 (0.46)	0.73
(WRPD)	Personal Characteristics	4.26 (0.51)	(WRPD)	Personal Characteristics	3.48 (0.40)	0.78
(IC)	Therapeutic communication participatory decision making	4.14 (0.58)	(PNP)	Specific critical thinking	3.43 (0.69)	0.71
(PNP)	Professional critical thinking (Using the nursing process)	4.11 (0.61)	(WRPD)	Legality	3.37 (0.72)	0.74
(PNP)	Specific critical thinking	4.11 (0.67)	(WRPD)	Mentoring	3.27 (0.70)	0.84
(NA)	Management & leadership	4.00 (0.55)	(NAC)	Management & leadership	3.15 (0.36)	0.85
(WRPD)	Mentoring	3.95 (0.65)	(PNP)	Professional critical thinking	3.14 (0.47)	0.81
(EBC)	Knowledge acquisition	3.94 (0.63)	(PNP)	General critical thinking	3.09 (0.98)	0.85
(IC)	Holism	3.70 (0.71)	(EBC)	Knowledge acquisition	2.90 (0.63)	0.80
(EBC)	Critical appraisal of evidence and implementing of applicable evidence	3.54 (0.63)	(ICC)	Holism	2.81(1.20)	0.73
(PNP)	General critical thinking	3.53 (0.64)	(EBC)	Critical appraisal of evidence and implementing of applicable evidence	2.59 (0.60)	0.94

Core competencies domains: EBC: Evidence-based nursing care, IC: Individualized care, NA: Nursing Administration, PNP: Professional nursing process, WRPD: Work readiness and professional development

The analysis of core competencies achieved and expected from both students’ and nursing faculty members’ perspectives revealed that, firstly, there was a significant difference between the mean scores of the two groups in all five core competencies (*P* < .001) and that the highest mean difference was related to evidence-based care with mean diff = 1 and the lowest mean difference was related to professional care process with mean diff = 0.70 (Table 4).

**Table 4:** Comparison of the Core competencies achieved from the student’s perspective and the expected Core competencies from the nursing faculty member’s perspective*

Core competencies categories	Groups	Mean (SD)	Mean diff (CG**)	*P*-value	CI 95%	
					Lower	Upper
Individualized care (IC)	Faculty	4.13 (0.44)	0.73	< 0.001	0.68	0.79
	Student	3.40 (0.47)				
Evidence-based nursing care (EBC)	Faculty	3.74 (0.57)	1.00	< 0.001	0.93	1.07
	Student	2.74 (0.55)				
Professional nursing process (PNP)	Faculty	3.92 (0.50)	0.70	< 0.001	0.63	0.76
	Student	3.21 (0.52)				
Nursing administration (NA)	Faculty	4.24 (0.42)	0.85	< 0.001	0.80	0.90
	Student	3.39 (0.33)				
Work readiness and professional development (WRPD)	Faculty	4.30 (0.45)	0.76	< 0.001	0.71	0.82
	Student	3.54 (0.39)				

*Independent t-test, **Competency Gap

Table 5 indicates that there was a significant difference between the mean scores achieved by students and nursing faculty members in all 5 core competencies and 17 sub-core Competencies (*p* < .001).

**Table 5:** Comparison of the Sub-Core competencies achieved from the student’s perspective and the expected Core competencies from the nursing faculty member’s perspective*

Core competencies categories	Sub-themes	Groups	Mean (SD)	Mean diff (CG**)	*P*-value	CI 95%	
						Lower	Upper
Individualized care	Cultural humility	Faculty	4.36 (0.48)	0.81	< 0.001	0.74	0.88
		Student	3.55 (0.70)				
	Value and ethical codes	Faculty	4.33 (0.46)	0.66	< 0.001	0.60	0.72
		Student	3.67 (0.47)				
	Holism	Faculty	3.70 (0.71)	0.83	< 0.001	0.77	1.00
		Student	2.81 (1.20)				
	Therapeutic communication participatory decision making	Faculty	4.14 (0.58)	0.59	< 0.001	0.52	0.86
		Student	3.55 (0.46)				
Evidence-based nursing care	Knowledge acquisition	Faculty	3.94 (0.63)	1.05	< 0.001	0.97	1.12
		Student	2.90 (0.63)				
	Critical appraisal of evidence and implementing of applicable evidence	Faculty	3.54 (0.63)	0.95	< 0.001	0.87	1.03
		Student	2.59 (0.60)				
Professional nursing process	General critical thinking	Faculty	3.53 (0.64)	0.44	< 0.001	0.35	0.54
		Student	3.09 (0.98)				
	Specific critical thinking	Faculty	4.11 (0.67)	0.68	< 0.001	0.60	0.77
		Student	3.43 (0.69)				
	Professional critical thinking (Using the nursing process)	Faculty	4.11 (0.61)	0.97	< 0.001	0.90	1.04
		Student	3.14 (0.47)				
Nursing administration	Management & leadership	Faculty	4.00 (0.55)	0.86	< 0.001	0.79	0.92
		Student	3.15 (0.36)				
	Informatics & documentation	Faculty	4.48 (0.44)	0.85	< 0.001	0.79	0.90
		Student	3.63 (0.49)				
Work readiness and professional development	Personal Characteristics	Faculty	4.26 (0.51)	0.76	< 0.001	0.72	0.84
		Student	3.48 (0.40)				
	Legality	Faculty	4.38 (0.64)	1.00	< 0.001	0.92	1.09
		Student	3.37 (0.72)				
	Clinical/Procedural skills	Faculty	4.28 (0.54)	0.61	< 0.001	0.55	0.68
		Student	3.67 (0.51)				
	Safety	Faculty	4.51 (0.45)	0.74	< 0.001	0.68	0.80
		Student	3.77 (0.51)				
	Preventative health services	Faculty	4.44 (0.63)	0.75	< 0.001	0.67	0.83
		Student	3.69 (0.60)				
	Mentoring	Faculty	3.95 (0.65)	0.68	< 0.001	0.59	0.76
		Student	3.27 (0.70)				

*Independent t-test, **Competency Gap

## Discussion

The study aimed to determine the difference between nursing students’ self-perceived level of competence and the level of competence expected of them by their nursing faculty members. The study results indicate that students scored highest in work readiness and professional development. However, they were not independent in this competency and required support. The National League for Nursing (NLN) recognizes nursing professional development as the goal of nursing education programs [47] However, Aguayo-Gonzalez [48] believes that the appropriate time for professional development is after entering a clinical setting. This theme includes personal characteristics, legality, clinical/ procedural skills, patient safety, preventive health services, and mentoring competence. Personality traits of nursing students are strong predictors of coping with nursing stress, as suggested by Imus [49]. These outcomes reflect changes in students’ individual characteristics during their nursing education. Personality changes, such as the need for patience and persistence in nursing care and understanding the nurse identity prepare students for the nursing profession, which is consistent with the studies of Neishabouri et al. [50]. Although the students demonstrated a higher level of competence in this theme, an examination of the items indicates that they can still not adapt to the challenges of bedside nursing and to use coping techniques. This presents a concerning issue that requires attention and resolution. Previous studies have shown that nursing education can be a very stressful experience [51–53].

Of course, there is no consensus on the definition of professionalism and the results of studies in this field are different. For example, Akhtar et al. (2013) identified common viewpoints about professionalism held by nursing faculty and students, and four viewpoints emerged humanists, portrayers, facilitators, and regulators [54]. The findings of another study showed that nursing students perceived vulnerability, symbolic representation, role modeling, discontent, and professional development are elements that show their professionalism [55]. The differences indicate that there may be numerous contextual variables that affect individuals’ perceptions of professionalism.

The legal aspects of nursing were the next item in this theme that students needed help with. The findings of studies regarding the legal competence of newly graduated nursing students are contradictory reported that only one-third of nurse managers were satisfied with the legal competence of newly graduated nursing students [56, 57]. Whereas the other studies showed that legality was the highest acquired competence for newly graduated nursing students [58, 59]. However, the results of this study indicated that legality may be a challenge for newly graduated nursing students. Benner [43] highlighted the significant change for new graduates in that they now have full legal and professional responsibility for the patient. Tong and Epeneter [60] also reported that facing an ethical dilemma is one of the most stressful factors for new graduates. Therefore, the inexperience of new graduates cannot reduce the standard of care that patients expect from them [60]. Legal disputes regarding the duties and responsibilities of nurses have increased with the expansion of their roles. This is also the case in Iran. Nurses are now held accountable by law for their actions and must be aware of their legal obligations. To provide safe healthcare services, it is essential to know of professional, ethical, and criminal laws related to nursing practice. The nursing profession is accountable for the quality of services delivered to patients from both professional and legal perspectives. Therefore, it is a valuable finding that nurse managers should support new graduates to better deal with ethical dilemmas. Strengthening ethical education in nursing schools necessitates integrating real cases and ethical dilemmas into the curriculum. Especially, Nursing laws are missing from Iran’s undergraduate nursing curriculum. By incorporating authentic case studies drawn from clinical practice, nursing schools provide students with opportunities to engage in critical reflection, ethical analysis, and moral deliberation. These real cases challenge students to apply ethical principles to complex and ambiguous situations, fostering the development of ethical competence and moral sensitivity. Furthermore, ethical reflection and debriefing sessions during clinical experiences enable students to discuss and process ethical challenges encountered in practice, promoting self-awareness, empathy, and professional growth. Overall, by combining theoretical instruction with practical application and the use of real cases, nursing schools can effectively prepare future nurses to navigate ethical dilemmas with integrity and compassion.

However, the theme of evidence-based nursing care was the lowest scoring, indicating that students need help with this theme. The findings from studies conducted in this field are varied. A limited number of studies reported that nursing students were competent to implement evidence-based care [61], while other researchers reported that nursing students’ attitudes toward evidence-based care to guide clinical decisions were largely negative [20, 62]. The principal barriers to implementing evidence-based care are lack of authority to change patient care policy, slow dissemination of evidence and lack of time at the bedside to implement evidence [10], and lack of knowledge and awareness of the process of searching databases and evaluating research [63]. While the European Higher Education Area (EHEA) framework and the International Council of Nurses Code of Ethics introduce the ability to identify, critically appraise, and apply scientific information as expected learning outcomes for nursing students [64, 65], the variation in findings highlights the complexity of the concept of competence and its assessment [23]. Evidence-Based Nursing (EBN) education for nursing students is most beneficial when it incorporates a multifaceted approach. Interactive workshops play a crucial role, providing students with opportunities to critically appraise research articles, identify evidence-based practices, and apply them to clinical scenarios. Simulation-based learning further enhances students’ skills by offering realistic clinical experiences in a safe environment. Additionally, clinical rotations offer invaluable opportunities for students to observe and participate in evidence-based practices under the guidance of experienced preceptors. Journal clubs foster a culture of critical thinking and ongoing learning, where students regularly review and discuss current research articles. Access to online resources such as databases and evidence-based practice guidelines allows students to stay updated on the latest evidence and best practices. To bridge the gap between clinical practice and academic theory, collaboration between nursing schools and healthcare institutions is essential. This collaboration can involve partnerships to create clinical learning environments that prioritize evidence-based practice, inter professional education activities to promote collaboration across disciplines, training and support for clinical preceptors, and continuing education opportunities for practicing nurses to strengthen their understanding and application of EBN [66]. By implementing these strategies, nursing education programs can effectively prepare students to become competent practitioners who integrate evidence-based principles into their clinical practice, ultimately improving patient outcomes.

The study’s findings regarding the second objective showed that nursing faculty members expected students to achieve the highest level of competence in work readiness and professional development, and the lowest in evidence-based nursing care competence. The results of the studies in this area revealed that there is a lack of clarity about the level of competence of newly graduated nursing students and that confusion about the competencies expected of them has become a major challenge [13, 67]. Evidence of nurse managers’ perceptions of newly graduated nursing student’s competence is limited and rather fragmented. There is a clear need for rigorous empirical studies with comprehensive views of managers, highlighting the key role of managers in the evaluation of nurse competence [1, 9]. Some findings also reported that nursing students lacked competence in primary and specialized care after entering a real clinical setting [68] and that nursing managers were dissatisfied with the competence of students [30].

The results of the present study on the third objective confirmed the gap between expected and achieved competence requirements. The highest average difference was related to evidence-based nursing care, and the lowest mean difference was related to the professional nursing process. The findings from studies in this field vary. For instance, Brown and Crookes [13] reported that newly graduated nursing students were not independent in at least 26 out of 30 competency domains. Similar studies have also indicated that nursing students need a structured program after graduation to be ready to enter clinical work [30]. It can be stated that the nursing profession does not have clear expectations of the competencies of newly graduated nursing students, and preparing them for entry into clinical practice is a major challenge for administrators [13]. These findings can be explained by the Duchscher transition shock [69]. It is necessary to support newly graduated nursing students to develop their competence and increase their self-confidence.

The interesting but worrying finding was the low expectations of faculty members and the low scores of students in the theme of evidence-based care. However, nursing students need to keep their competencies up to date to provide safe and high-quality care. The WHO also considers the core competencies of nurse educators to be the preparation of effective, efficient, and skilled nurses who can teach the evidence-based learning process and help students apply it clinically [44]. The teaching of evidence-based nursing care appears to vary across universities, and some clinical Faculties do not have sufficient knowledge to support students. In general, it can be stated that the results of the present study are in line with the context of Iran. Some of the problems identified include a lack of attention to students’ academic talent, a lack of a competency-based curriculum, a gap between theory and clinical practice, and challenges in teaching and evaluating the achieved competencies [42].

### Strengths and limitations

The study was conducted on a national level with a sizable sample. It is one of the first studies in Iran to address the gap between students’ self-perceived competence levels and nursing faculty members’ expected competency levels. Nevertheless, one of the limitations of the study is the self-report nature of the questionnaire, which may lead to social desirability bias. In addition, the COVID-19 pandemic coinciding with the student’s first and second years could potentially impact their educational quality and competencies. The limitations established during the outbreak negatively affected the nursing education of students worldwide.

## Conclusion

Acquiring nursing competencies is the final product of nursing education. The current study’s findings suggest the existence of an academic-practice gap, highlighting the need for educators, faculty members, and nursing managers to collaborate in bridging the potential gap between theory and practice. While nursing students were able to meet some expectations, such as value and ethical codes, there is still a distance between expectations and reality. Especially, evidence-based care was identified as one of the weaknesses of nursing students. It is recommended that future research investigates the best teaching strategies and more objective assessments of competencies. The findings of this study can be used as a guide for the revision of undergraduate nursing education curricula, as well as a guide for curriculum development based on the development of competencies expected of nursing students. Nursing managers can identify existing gaps and plan to fill them and use them for the professionalization of students. This requires the design of educational content and objective assessment tools to address these competencies at different levels throughout the academic semester. This significant issue necessitates enhanced cooperation between healthcare institutions and nursing schools. Enhancing nursing education requires the implementation of concrete pedagogical strategies to bridge the gap between theoretical knowledge and practical skills. Simulation-based learning emerges as a pivotal approach, offering students immersive experiences in realistic clinical scenarios using high-fidelity simulators [70]. Interprofessional education (IPE) is also instrumental, in fostering collaboration among healthcare professionals and promoting holistic patient care. Strengthening clinical preceptorship programs is essential, with a focus on providing preceptors with formal training and ongoing support to facilitate students’ clinical experiences and transition to professional practice [71]. Integrating evidence-based practice (EBP) principles throughout the curriculum cultivates critical thinking and inquiry skills among students, while technology-enhanced learning platforms offer innovative ways to engage students and support self-directed learning [72]. Diverse and comprehensive clinical experiences across various healthcare settings ensure students are prepared for the complexities of modern healthcare delivery. By implementing these practical suggestions, nursing education programs can effectively prepare students to become competent and compassionate healthcare professionals.

## Data Availability

The data that support the findings of this study are available from the corresponding author upon reasonable request.
